# Case Report: Thoracic spinal metastasis without definite intrahepatic recurrence after conversion therapy and hepatectomy for hepatocellular carcinoma

**DOI:** 10.3389/fonc.2026.1931013

**Published:** 2026-09-09

**Authors:** Jiahui Chen, Yanhua Huang, Difan Zhou

**Affiliations:** 1 Department of Pathology, Shaoxing People’s Hospital, Shaoxing, Zhejiang, China; 2 School of Medicine, Shaoxing University, Shaoxing, Zhejiang, China; 3 Department of Ultrasound, Shaoxing People’s Hospital, Shaoxing, Zhejiang, China; 4 Department of Hepatobiliary and Pancreatic Surgery, Shaoxing People’s Hospital, Shaoxing, Zhejiang, China

**Keywords:** alpha-fetoprotein, case report, des-gamma-carboxy prothrombin, hepatocellular carcinoma, metastatic spinal cord compression

## Abstract

**Introduction:**

Conversion therapy followed by hepatectomy can achieve intrahepatic control in selected patients with hepatocellular carcinoma (HCC), but occult extrahepatic progression may be missed when surveillance remains centered on the liver.

**Results:**

A 61-year-old man with hepatitis B-related multifocal HCC underwent transarterial chemoembolization, lenvatinib, four documented preoperative cycles of tislelizumab, and subsequent hepatectomy. Pathology showed treatment-related response, negative margins, and no microvascular invasion. During scheduled surveillance, alpha-fetoprotein and des-gamma-carboxy prothrombin increased progressively despite no definite intrahepatic recurrence on available liver imaging. PET/CT later revealed a destructive T2 vertebral lesion. After local radiotherapy, no dedicated spine MRI or multidisciplinary spinal assessment was performed before neurological deterioration. At emergency admission, lower-limb strength was initially preserved on the right and partially reduced on the left, followed by rapid bilateral paralysis. Contrast-enhanced thoracic MRI showed multilevel metastases with a dominant T8 lesion causing epidural spinal cord compression and pathological fracture. Fixation and decompression relieved radiological compression, but severe motor and autonomic dysfunction persisted.

**Discussion:**

The clinical value of this case lies in a surveillance gap rather than rarity alone. Persistent AFP/DCP elevation with negative liver imaging should prompt systemic restaging for occult extrahepatic disease. Once a destructive vertebral lesion is detected, early spine MRI and multidisciplinary assessment of stability and epidural compression are warranted before irreversible neurological injury develops.

## 1 Introduction

Treatment for hepatocellular carcinoma (HCC) has changed markedly with the broader use of locoregional therapy, targeted agents, immune checkpoint inhibitors, and conversion surgery. In carefully selected patients, transarterial chemoembolization (TACE) combined with systemic therapy can reduce intrahepatic tumor burden and make hepatectomy possible, with apparently favorable local control in some cases. As survival improves, extrahepatic progression has become increasingly relevant during follow-up. Bone is a recognized site of HCC metastasis, and spinal involvement is particularly important because it may cause pain, mechanical instability, pathological fracture, epidural spinal cord compression, neurological decline, and loss of independence (1–4).

In the present case, the key issue was not the rarity of thoracic bone metastasis alone, but the clinical challenge of recognizing extrahepatic progression after effective intrahepatic control. Extrahepatic recurrence is recognized after curative-intent treatment for HCC (5), and isolated bone metastases have been identified on PET/CT (6). This case was notable for the discordance between progressively increasing AFP/DCP levels and the absence of definite intrahepatic recurrence after conversion therapy and hepatectomy. The disease subsequently progressed from an initially detected T2 vertebral lesion to multilevel thoracic spinal metastases, with T8 becoming the dominant symptomatic level responsible for metastatic spinal cord compression. We present this case in accordance with the CARE reporting checklist.

## 2 Case description

A 61-year-old Han Chinese man with chronic hepatitis B-related cirrhosis was diagnosed with multifocal right-lobe hepatocellular carcinoma (HCC) in September 2024. Baseline contrast-enhanced MRI and CT showed arterial-phase hyperenhancement and portal venous washout; the largest lesion measured approximately 35 × 23 mm on MRI and 33 × 35 mm on CT. No definite macrovascular invasion, ascites, or enlarged retroperitoneal lymph nodes were identified, and the disease was classified as China Liver Cancer stage IIb. Chest CT showed scattered indeterminate tiny pulmonary nodules that remained stable on November 29, 2024 and were not considered definite metastases. PET/CT and bone scintigraphy were not performed at diagnosis because there were no skeletal symptoms or suggestive imaging findings. ECOG performance status was 0. Liver function was Child–Pugh class A and ALBI grade 2, with albumin 35.9 g/L, total bilirubin 13 μmol/L, normal PT/INR, and no ascites or hepatic encephalopathy. HBV DNA was 2.14 × 10³ IU/mL, and entecavir was administered during subsequent treatment.

**Figure 1:**
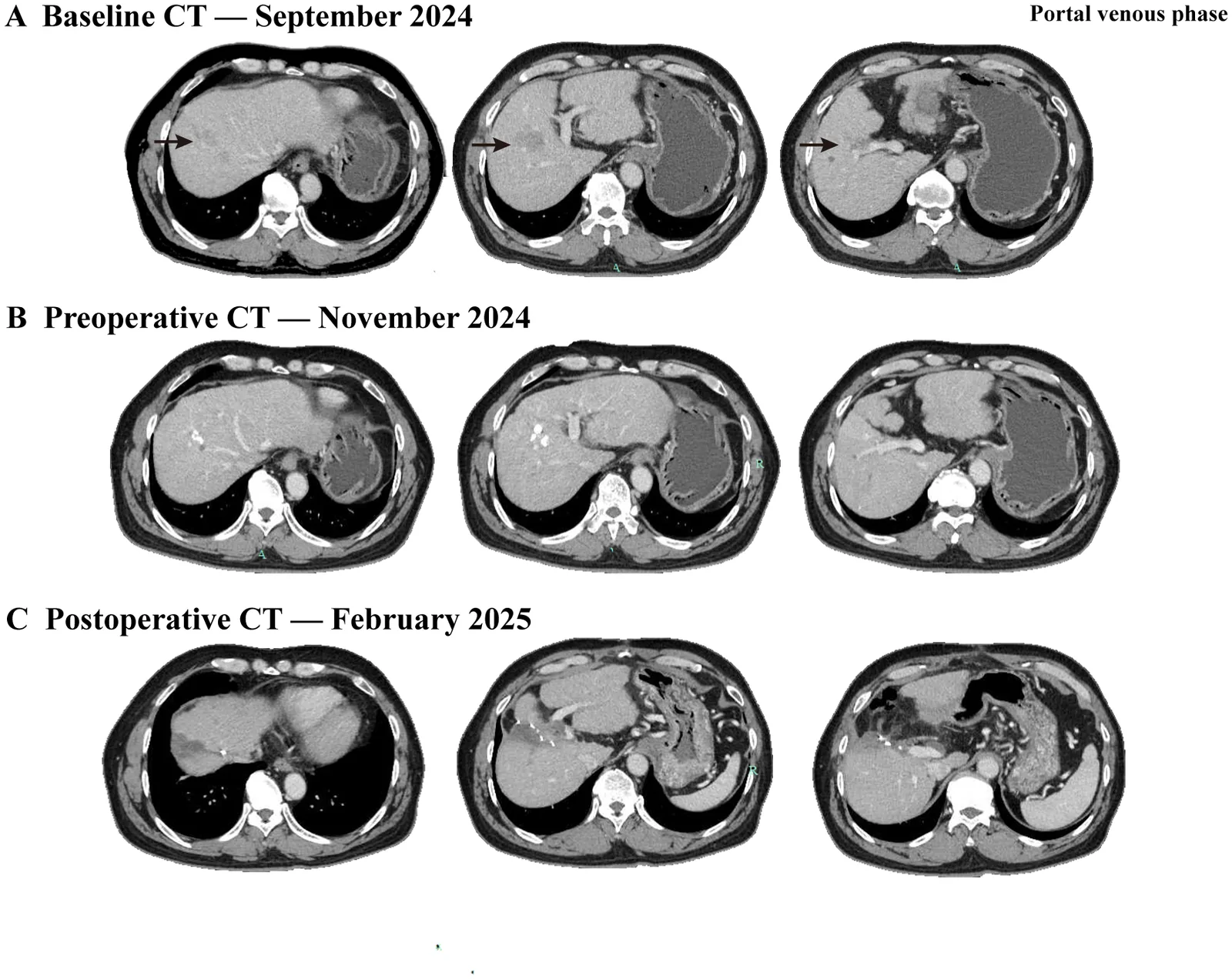
Longitudinal CT findings before and after hepatectomy. **(A)** Baseline portal venous-phase CT in September 2024 showing multifocal tumors in the right hepatic lobe. **(B)** Preoperative CT in November 2024 after TACE, lenvatinib, and four cycles of tislelizumab, showing tumor regression and intratumoral lipiodol deposition. **(C)** Surveillance CT on February 18, 2025, showing postoperative changes without definite intrahepatic recurrence.

Conversion therapy comprised TACE followed by lenvatinib 8 mg once daily and tislelizumab 200 mg every 21 days from September 20, 2024. Four preoperative cycles of tislelizumab were clearly documented. Response was assessed according to RECIST version 1.1. After approximately 3 months, contrast-enhanced CT showed reduced tumor burden, marked lipiodol deposition, no new measurable lesions, and no definite extrahepatic progression, accompanied by a marked decrease in AFP. Multidisciplinary reassessment supported conversion surgery based on the favorable radiological and biochemical response, absence of definite macrovascular invasion or extrahepatic disease, preserved liver function, and ECOG performance status of 0 (**Figure 1**).

Hepatectomy and cholecystectomy were performed on December 6, 2024. Pathological examination showed a 3.2 × 2.5 × 1.7-cm tumor bed containing moderately to poorly differentiated HCC with partial necrosis and treatment-related changes. Microvascular and capsular invasion were absent, the surgical margin was negative, and the background liver showed nodular cirrhosis. Because the original pathology report did not quantify viable tumor or necrosis, their exact percentages could not be determined retrospectively. Postoperative hepatic arteriography and TACE were performed on January 17, 2025, after which systemic therapy was continued.

**Figure 2:**
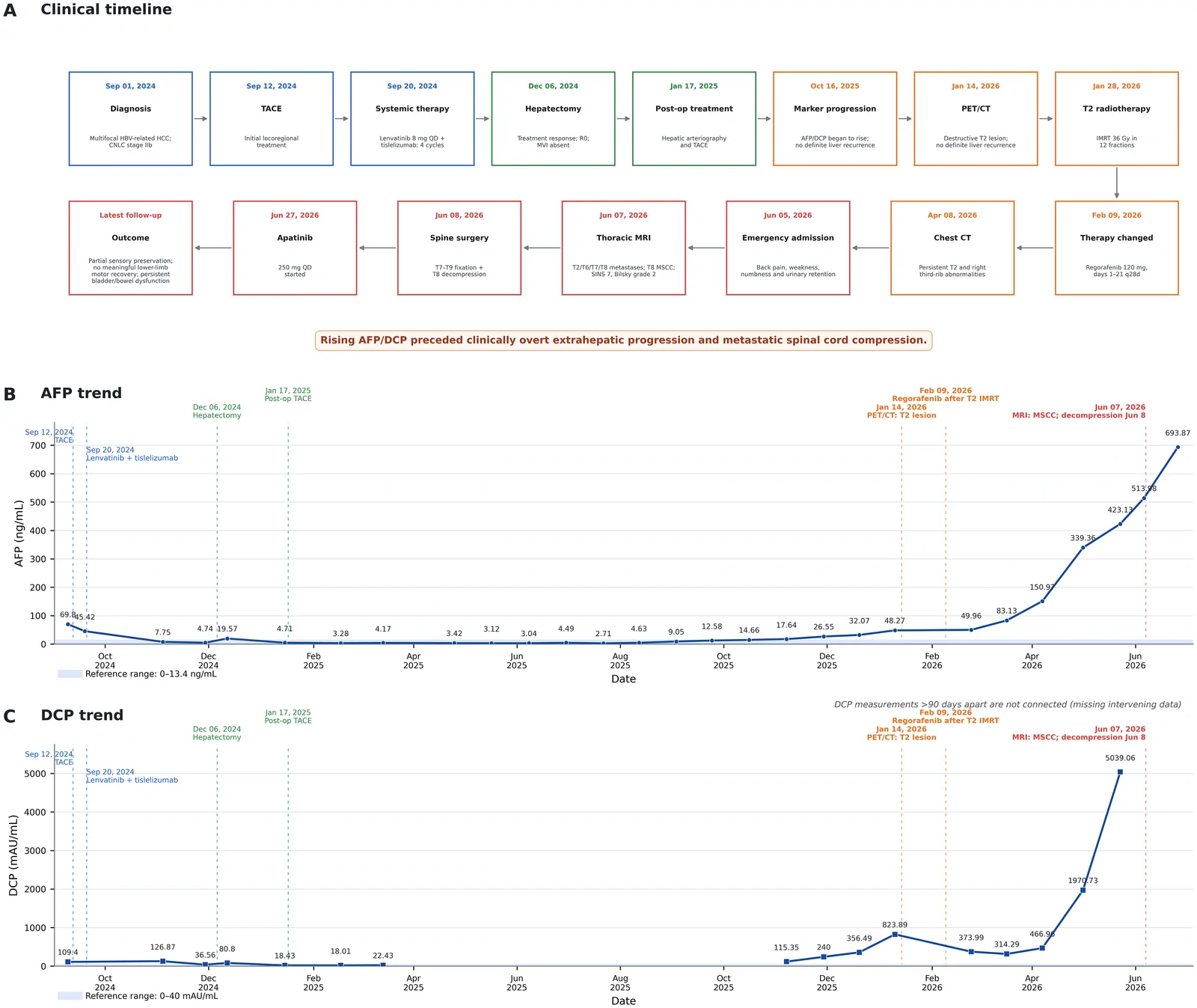
Clinical timeline and serial AFP/DCP trends. **(A)** Clinical timeline from HCC diagnosis and conversion therapy through hepatectomy, postoperative treatment, biomarker progression, vertebral metastasis, neurological deterioration, spine surgery, and follow-up. The timeline continues from the upper to the lower row. **(B)** Serial serum alpha-fetoprotein (AFP) concentrations. **(C)** Serial des-gamma-carboxy prothrombin (DCP) concentrations. Shaded areas indicate the institutional reference ranges (AFP, 0–13.4 ng/mL; DCP, 0–40 mAU/mL). DCP values separated by more than 90 days are not connected because intervening measurements were unavailable.

Clinical and laboratory follow-up was performed at each 21-day treatment cycle, including serial AFP and DCP measurements. Intrahepatic surveillance was conducted approximately every 3 months using alternating contrast-enhanced CT and MRI. AFP began to rise approximately 10 months after hepatectomy, followed by DCP approximately 1 month later. The institutional reference ranges were 0–13.4 ng/mL for AFP and 0–40 mAU/mL for DCP. Both markers subsequently increased despite no definite intrahepatic recurrence on serial liver imaging (**Figure 2**).

**Figure 3:**
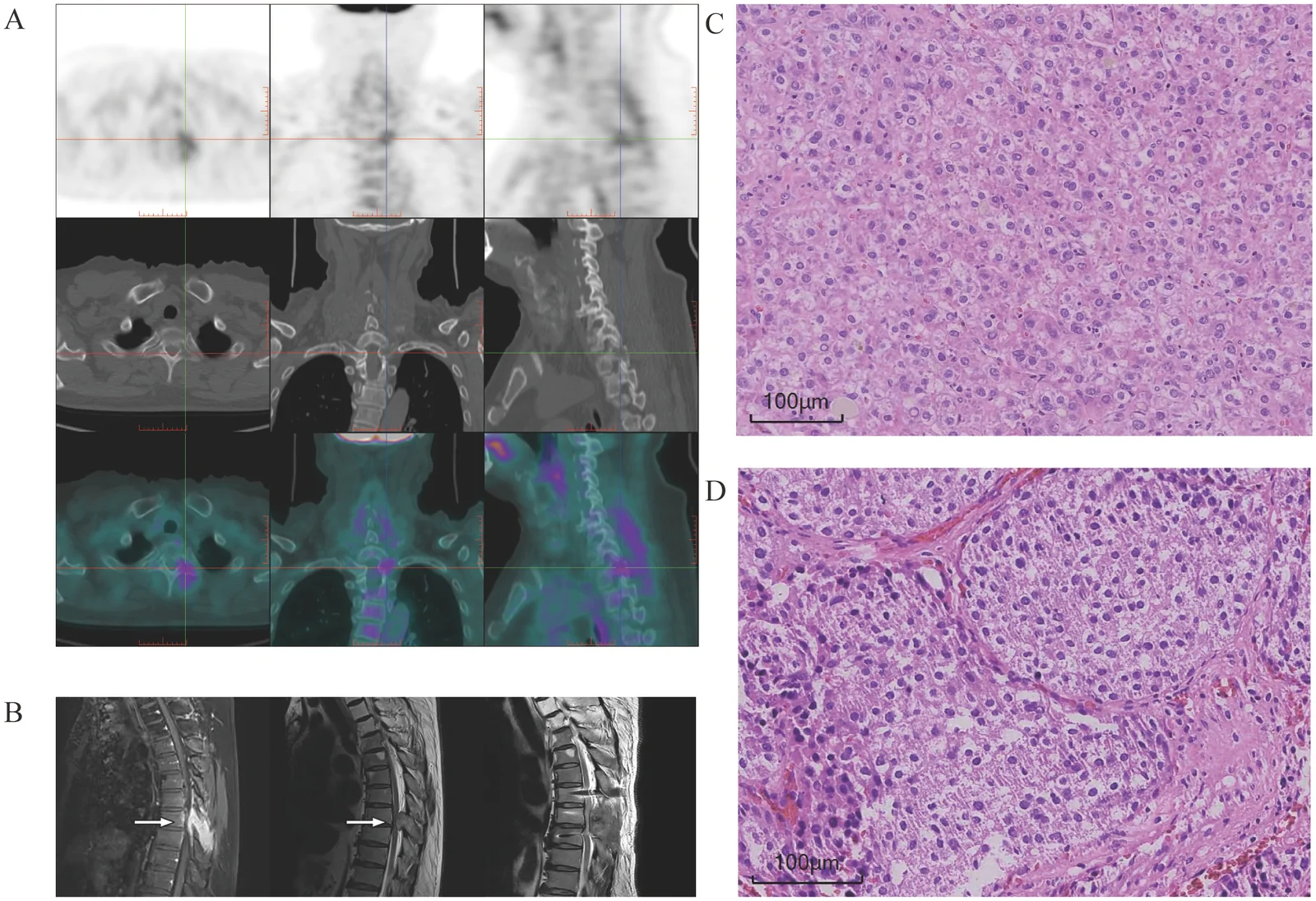
Spinal metastasis, cord compression, and histopathological confirmation. **(A)** ^18^F-FDG PET/CT showing a destructive FDG-avid T2 vertebral lesion suspicious for metastasis. **(B)** Thoracic MRI showing multilevel metastases and a dominant T8 lesion with epidural spinal cord compression; postoperative MRI confirmed decompression and fixation. **(C)** Primary hepatocellular carcinoma showing nested and trabecular growth with treatment-related changes. **(D)** Spinal metastatic hepatocellular carcinoma involving fibrous tissue and bone. Both **(C, D)** are hematoxylin and eosin-stained sections; scale bars are shown in the images.

Whole-body ^18^F-FDG PET/CT on January 14, 2026 showed no definite recurrence in the hepatic resection or treatment region but revealed a destructive FDG-avid lesion involving the left lamina and transverse process of T2, suspicious for bone metastasis. An additional abnormality was noted in the right anterior third rib (**Figure 3A**). The patient received intensity-modulated radiotherapy to the T2 lesion (36 Gy in 12 fractions). Lenvatinib was subsequently replaced with regorafenib 120 mg once daily on days 1–21 of each 28-day cycle from February 9, 2026. DCP decreased transiently, but both AFP and DCP later increased again.

No dedicated thoracic spine MRI was performed before radiotherapy, and no follow-up spine MRI, whole-spine MRI, repeat PET/CT, bone scintigraphy, or formal spine surgical consultation was performed before emergency admission. Chest CT on April 8, 2026 showed stable tiny pulmonary nodules but persistent bony abnormalities at T2 and the right anterior third rib. No subsequent spine-focused imaging or multidisciplinary spinal assessment was performed.

On June 5, 2026, the patient presented with left lower-limb numbness lasting 2 days and worsening markedly approximately 1 hour before admission, accompanied by dysuria and urinary retention. At that time, comprehensive intrahepatic restaging with contrast-enhanced liver CT or MRI was not performed; therefore, intrahepatic disease status at this exact time point could not be definitively determined. Initial lower-limb strength was 5/5 on the right and 4−/5 on the left, followed by rapid progression to bilateral paralysis. By contrast-enhanced thoracic MRI on June 7, motor and sensory function below T8 had been lost and urinary retention persisted, consistent with Frankel grade A paraplegia. MRI demonstrated metastases involving T2, T6, T7, and T8, with a dominant T8 lesion causing intraspinal extension, pathological fracture, and spinal cord compression. Dexamethasone 10 mg was administered by intravenous push preoperatively.

Posterior T7–T9 fixation, T8 laminectomy and decompression, and nerve-root adhesiolysis were performed on June 8, 2026. Postoperative MRI on June 17 confirmed internal fixation and relief of spinal cord compression (**Figure 3B**). Pathological examination of the spinal lesion showed malignant tumor cells infiltrating fibrous tissue and bone (**Figures 3C, D**). Immunohistochemistry supported metastatic HCC: Glypican-3 and AFP were partially positive, Arginase-1 was positive, Hepatocyte was focally positive, the Ki67 index was approximately 30%, and CK7 and CK19 were negative.

## 3 Diagnostic assessment

The diagnostic assessment integrated serial intrahepatic surveillance, tumor-marker kinetics, whole-body metabolic imaging, spine MRI, neurological grading, and pathological confirmation. Available follow-up liver imaging before emergency admission did not show definite intrahepatic recurrence, whereas AFP and DCP increased progressively. Whole-body ^18^F-FDG PET/CT identified a metabolically active destructive T2 vertebral lesion, raising suspicion for extrahepatic skeletal progression. Subsequent contrast-enhanced thoracic spine MRI showed more extensive thoracic spinal disease and identified T8 as the dominant symptomatic level causing epidural spinal cord compression. Retrospective review of the June 7 MRI yielded a Spinal Instability Neoplastic Score (SINS) of 7, indicating potential instability, and a Bilsky epidural spinal cord compression grade of 2. At the time of MRI assessment, the patient had Frankel grade A paraplegia and urinary retention. Differential considerations included bone metastasis, post-radiotherapy change, inflammatory disease, and other bone lesions. The combined imaging pattern, rapid neurological progression, and histopathological and immunohistochemical findings supported metastatic HCC with spinal cord compression.

## 4 Therapeutic intervention

The patient underwent TACE on September 12, 2024. Angiography demonstrated multiple tumor-staining lesions in the right hepatic lobe. After selective microcatheterization, intra-arterial oxaliplatin 100 mg, floxuridine 500 mg, epirubicin 30 mg, and iodized oil 4 mL were administered, followed by gelatin sponge embolization. Lenvatinib 8 mg once daily plus tislelizumab 200 mg every 21 days was initiated on September 20, 2024. Four preoperative cycles of tislelizumab were documented. Following hepatectomy, hepatic arteriography and postoperative TACE were performed on January 17, 2025, after which systemic therapy was continued.

After PET/CT identified a destructive T2 vertebral lesion suspicious for metastasis, the patient received intensity-modulated radiotherapy to the T2 lesion (36 Gy in 12 fractions). Lenvatinib was subsequently replaced with regorafenib 120 mg once daily on days 1–21 of each 28-day cycle, beginning February 9, 2026. Following rapid neurological deterioration, contrast-enhanced thoracic spine MRI was performed on June 7 to define the involved levels and guide operative planning. Posterior T7–T9 fixation, T8 laminectomy and decompression, and nerve-root adhesiolysis were performed on June 8. During postoperative rehabilitation, oral apatinib 250 mg once daily was initiated on June 27, 2026.

## 5 Follow-up and outcomes

Postoperative MRI confirmed radiological decompression of the spinal cord; however, severe neurological and functional impairment persisted during rehabilitation. The modified Barthel Index was 9, indicating marked dependence in activities of daily living. Upper-limb strength was preserved, whereas bilateral lower-limb strength remained 0/5. The neurological level of injury was T8, with absent voluntary anal contraction and persistent neurogenic bladder and bowel dysfunction. Subsequent spinal cord injury assessment was consistent with American Spinal Injury Association Impairment Scale (AIS) grade B, indicating sensory preservation below the neurological level without meaningful motor recovery in the lower limbs. Rehabilitation focused on preventing complications, maintaining joint mobility through passive range-of-motion exercises, managing neurogenic bladder and bowel dysfunction, facilitating wheelchair mobility, and maximizing independence in daily activities.

## 6 Patient perspective

According to the patient’s legal representative, the early back pain and lower-limb numbness were initially not recognized as possible signs of spinal metastasis. When urinary difficulty and progressive weakness developed, neurological deterioration occurred rapidly and caused major distress to the patient and family. After surgery, the family understood that decompression had relieved spinal cord compression on imaging, but that neurological recovery might be incomplete. They hoped that other patients with HCC would seek early medical attention if persistent back pain, limb numbness, urinary symptoms, or rising tumor markers occurred.

## 7 Discussion

This case illustrates the clinical challenge of recognizing extrahepatic progression after effective intrahepatic control of hepatocellular carcinoma (HCC). The patient initially achieved a favorable response to TACE combined with lenvatinib and tislelizumab, followed by hepatectomy with negative margins, absence of microvascular invasion, and treatment-related pathological changes. However, AFP and DCP subsequently increased despite no definite intrahepatic recurrence on serial liver imaging. The later detection of vertebral metastases and metastatic spinal cord compression demonstrates that extrahepatic progression may occur even when intrahepatic disease appears controlled (1, 2).

The 2023 AASLD Practice Guidance recommends cross-sectional imaging of the abdomen and chest together with serum AFP every 3–6 months after surgical resection, with indefinite surveillance because the optimal timing and duration remain uncertain (7). DCP was also monitored in this patient but is not included in the cited AASLD recommendation. AFP and DCP were measured at each treatment cycle, and liver CT or MRI was performed approximately every 3 months. This strategy identified progressive biomarker elevation despite negative intrahepatic imaging. The discordance between tumor-marker kinetics and liver imaging highlights the importance of considering occult extrahepatic disease when AFP or DCP continues to increase without an identifiable intrahepatic source.

Bone metastasis has become increasingly relevant as survival improves with locoregional treatment, systemic therapy, immunotherapy, and conversion surgery. Spinal involvement is particularly important because it may cause mechanical pain, instability, pathological fracture, epidural extension, spinal cord compression, neurological deterioration, and loss of ambulation. Once severe neurological deficits such as urinary retention or paraplegia develop, functional recovery is often incomplete even after radiological decompression (1–4).

In this patient, PET/CT identified a destructive T2 vertebral lesion suspicious for metastasis, after which local radiotherapy was administered and lenvatinib was replaced with regorafenib. Dedicated spine MRI, whole-spine imaging, repeat PET/CT, bone scintigraphy, and formal spine surgical consultation were not performed before neurological deterioration. Follow-up chest CT continued to show thoracic skeletal abnormalities. Subsequent contrast-enhanced thoracic MRI demonstrated multilevel thoracic metastases, with T8 becoming the dominant symptomatic level responsible for epidural spinal cord compression. This clinical course illustrates the potential value of early spine-focused imaging and multidisciplinary assessment after detection of a destructive vertebral lesion, although it cannot be determined whether earlier intervention would have prevented paraplegia (3, 4, 8, 9).

This case supports a stepwise escalation strategy rather than routine whole-body imaging for every patient after HCC resection. When AFP or DCP rises persistently despite negative liver imaging, clinicians should first confirm the trend and assess the adequacy of intrahepatic imaging. Continued or accelerating elevation should prompt systemic restaging with chest and abdominal/pelvic cross-sectional imaging, consistent with the recommended scope of postoperative surveillance (7). PET/CT may be considered when conventional imaging remains unrevealing or skeletal symptoms are present because HCC bone metastases can be detected using PET/CT (6). Once a destructive vertebral lesion is identified, timely contrast-enhanced spine MRI and multidisciplinary evaluation are warranted to characterize epidural involvement, assess mechanical stability, and determine whether preventive or urgent intervention is required (3, 4, 8–10).

This report has limitations. It describes a single patient, and the proportions of viable tumor and necrosis in the hepatectomy specimen could not be determined retrospectively. In addition, dedicated spine MRI was not performed when the T2 lesion was initially identified. The retrospective nature of this report does not allow us to determine whether earlier spine MRI or intervention would have prevented paraplegia. Nevertheless, the case provides a clinically actionable lesson: persistent AFP or DCP elevation without definite intrahepatic recurrence should prompt consideration of extrahepatic progression, and a destructive vertebral lesion should lead to timely spine-focused imaging and multidisciplinary assessment.

## 8 Conclusion

Progressive AFP and DCP elevation after conversion therapy and hepatectomy for HCC may indicate occult extrahepatic progression, even when available liver imaging shows no definite intrahepatic recurrence. Persistent or accelerating marker elevation should prompt stepwise systemic restaging rather than liver-centered surveillance alone. In patients with suspected or known vertebral involvement, new back pain, neurological symptoms, or persistent skeletal abnormalities should trigger early spine MRI and multidisciplinary assessment of spinal stability and epidural compression before irreversible neurological deficits develop.

## Data Availability

The original contributions presented in the study are included in the article/supplementary material. Further inquiries can be directed to the corresponding authors.
